# Imaging of lumbar spinal surgery complications

**DOI:** 10.1007/s13244-015-0435-8

**Published:** 2015-10-02

**Authors:** Ajay Malhotra, Vivek B. Kalra, Xiao Wu, Ryan Grant, Richard A. Bronen, Khalid M. Abbed

**Affiliations:** Department of Diagnostic Radiology, Yale School of Medicine, Box 208042, Tompkins East 2, 333 Cedar St, New Haven, CT 06520-8042 USA; Department of Neurosurgery, Yale School of Medicine, New Haven, CT USA

**Keywords:** CT, MR, Lumbar spinal surgeries, Complications, Artefact

## Abstract

**Abstract:**

Lumbar spine surgery for spinal stenosis is a frequently performed procedure and was the fastest growing type of surgery in the US from 1980 to 2000. With increasing surgical invasiveness, postoperative complications also tend to be higher. Cross-sectional imaging techniques (CT and MRI) are more sensitive than radiographs and play an increasingly important role in evaluation of patients with lumbar spine surgery. Their use in patients with metallic implants is somewhat limited by artefacts, which can obscure pathology and decrease accuracy and reader confidence. Metal artefact reduction techniques have been developed, which can significantly improve image quality and enable early detection of postoperative complications. Complications can occur throughout postoperative course. Early complications include hardware displacement, incidental durotomy, postoperative collections—most commonly seroma, and less likely haematoma and/or infection. Incidental durotomy with CSF leak causing intracranial hypotension has characteristic MR brain findings and diagnosis of occult leak sites have been improved with use of dynamic CT myelography. Haematomas, even when compressing the thecal sac, are usually asymptomatic. Early infection, with nonspecific MR findings, can be diagnosed accurately using dual radiotracer studies. Delayed complications include loosening, hardware failure, symptomatic new or recurrent disc herniation, peri-/epidural fibrosis, arachnoiditis, and radiculitis.

**Teaching Points:**

• *CT and MRI play an increasingly important role in evaluation of patients with lumbar spine surgery*

• *Complications can occur throughout the postoperative course and early detection is critical*

• *Artefact reduction techniques can improve image quality for early and improved detection of complications*

## Introduction

Back pain is a frequent clinical complaint and affects 80 % of the population in their lifetime [1]. The rate of spinal surgery is increasing, although with significant geographic variation in spine surgery rates and spinal fusion rates [2, 3]. In a study evaluating trends associated with surgery for lumbar spinal stenosis in older adults, Deyo et al. (2010) found a 15-fold increase in rates of complex fusion procedures from 2002 to 2007, with higher rates of life-threatening complications (5.6 %) compared to simple decompression (2.3 %). A higher rate for rehospitalisation within 30 days was also noted with complex fusion (13 %) compared to decompression (7.8 %) [4].

Imaging through plain radiography, CT, MRI, and nuclear medicine is key to the evaluation of lumbar spinal postoperative patients. Imaging may be performed as a routine to evaluate the position and appearance of spinal instrumentation or to assess the progression of spinal fusion as well as to evaluate postoperative complications or in case of failed back syndrome. Advances in both CT and MR metallic artefact reduction have allowed for significantly improved assessment of the hardware and postoperative site. Plain radiography and CT are important in evaluating hardware malpositioning and loosening. CT, MR, and nuclear medicine have critical diagnostic roles in evaluation of infection and failed back surgery syndrome (FBSS) characterised by symptomatic new or recurrent disc herniation, peri-/epidural fibrosis, arachnoiditis, and radiculitis. Early complications include intracanalicular pedicle screw placement, incidental durotomy, haematoma, and infection. Delayed complications include loosening, hardware failure, symptomatic new or recurrent disc herniation, peri-/epidural fibrosis, arachnoiditis, and radiculitis.

## Artefacts

Hardware material composition and size affect both CT and MR artefacts. Titanium alloy is both less dense and less magnetic than stainless steel, resulting in less streak artefact from beam hardening on CT and less magnetic field distortion on MR. Metallic artefact is related to density, with less CT artefact resulting from less dense materials. Materials can be arranged in ascending artefact in the following order: plastic < titanium < vitallium < stainless steel < cobalt-chrome [5]. Less beam hardening occurs with stronger CT tube voltage, so images should be acquired at 120–140 kVp rather than 80 kVp, with the consequence of doubling of the radiation dose. Additionally CT acquisition parameters include high tube charge, lower pitch, and thin sections, also with the consequence of increased dose. Controlling CT post-processing parameters such as thicker sections, soft tissue instead of bony reconstruction kernels, and extended CT Hounsfield scale can further reduce artefacts. Advances in CT technology allow for higher currents, improving imaging in obese patients. Although not widely available yet, dual energy CT has significant potential to reduce metallic artefacts [6]. Sinogram inpainting methods have also been shown to reduce CT metallic artefact [7].

**Fig. 1:**
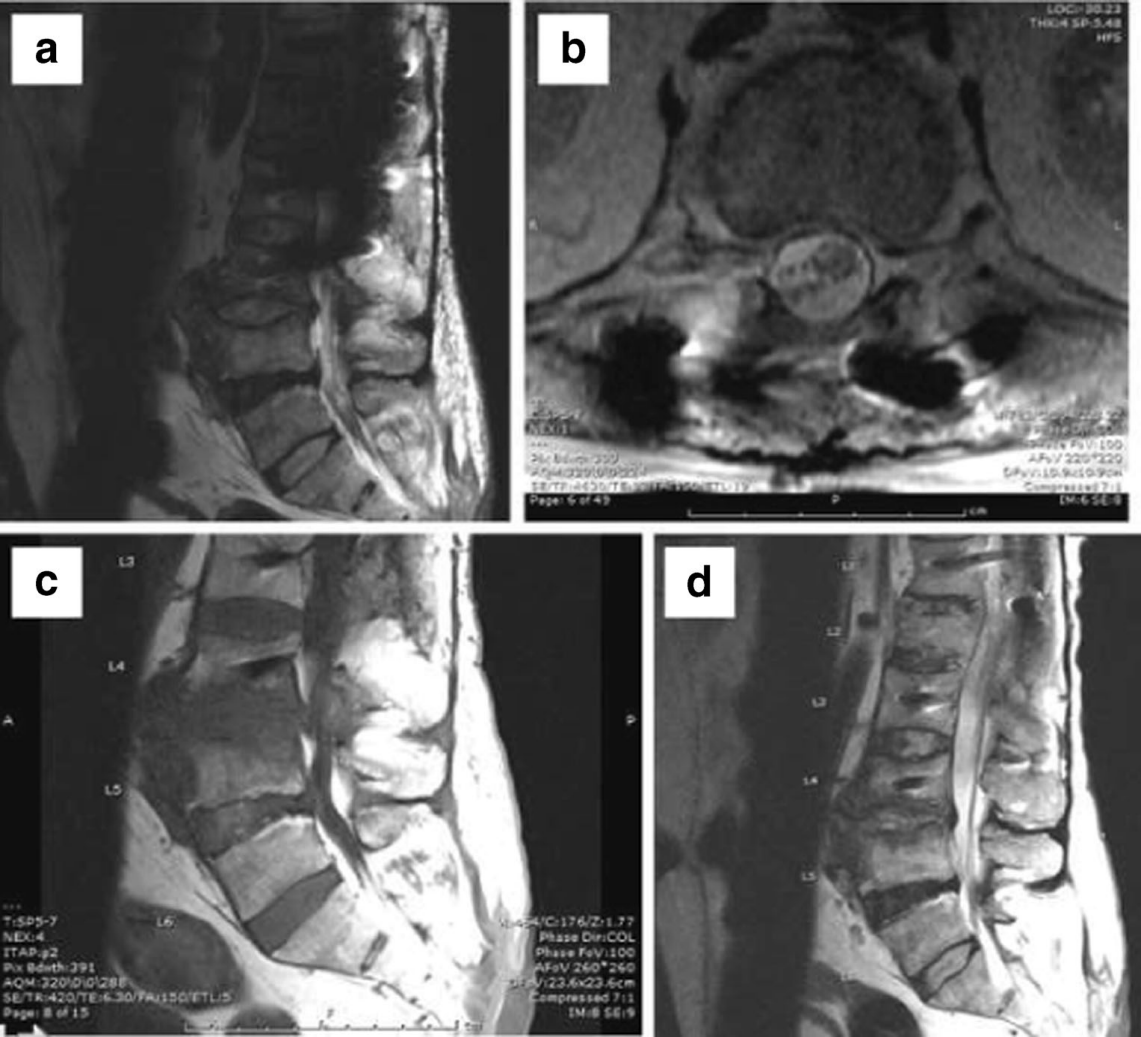
Scan performed at 3T (**a**) has significant susceptibility artefacts and image degradation. Optimized images at 1.5 T (**b**, **c**, and **d**) are of significantly improved image quality with good visualisation of the canal and neural foramina

MR may demonstrate metallic artefacts even when no hardware is placed, arising from tiny metallic drill bit fragments in postoperative beds. Susceptibility artefacts may result in a loss of signal in phase direction by intravoxel dephasing and spatial misregistration in the frequency encoding and slice selection gradient. Specific MR artefacts are related to hardware composition, orientation and shape, MR magnet field strength, and imaging sequence type and parameters. Since artefacts are significantly fewer when the hardware is perpendicular to the magnet, there are fewer artefacts caused by pedicle screws at the L1–L3 levels compared to L4–S1. Spherical implants result in greater artefacts than cylindrical ones [8]. Fast spin echo sequences have fewer artefacts than conventional spin echo or gradient echo sequences. Fat suppression with short inversion time inversion recovery (STIR) has fewer artefacts than frequency-selective fat saturation. Artefacts are proportional to the magnet strength, so imaging should be preferentially performed on 1.5-T scanners. However, higher gradient strengths and broader receiver bandwidths with newer coils can offset the greater artefact effect at 3.0 T. For any sequence, artefacts can be minimised by using a small field of view, high-resolution matrix, and thin sections (Fig. 1). Advanced artefact reduction techniques include view angle tilting, slice encoding for metal artefact correction, multi-acquisition variable-resonance image combination, single-point imaging, prepolarised imaging, and dual reversed-gradient acquisitions. View angle tilting corrects for intra-slice (in-plane) distortion and is used in combination with slice encoding for metal artefact correction, which corrects for adjacent slice (through-plane) distortions.

## Complications

The number of lumbar spinal surgery complications is proportional to the extent of surgery, lowest with minimally invasive degenerative disk procedures and greatest for scoliosis and dysraphism repairs. Complications can occur at any time in the postoperative period. Immediate postoperative complications are related to improper hardware placement, most commonly an intracanalicular pedicular screw course, resulting in vascular or neural injury. Complications that occur in the scale of days and weeks post hardware placement are seroma/haematoma and infection within the postoperative site, including the hardware or superinfection of postoperative collections. Hardware loosening usually occurs over a period of months to years post surgery and is associated with hardware failure.

Anterior lumbar interbody fusion (ALIF) is performed through a lower abdominal or retroperitoneal approach and specific complications include vascular injury and retrograde ejaculation. Venous laceration is more common than arterial injury and more common in laparoscopic procedures [9]. Visceral injury such as bowel perforation is rare [10]. Retrograde ejaculation due to manipulation of the autonomic plexus, or weakness of hip flexors with lateral/axial interbody fusion and damage to the lumbosacral plexus, does not require further imaging [11, 12].

### Improper hardware placement

Although not a true post-surgical complication, it is important to identify hardware misplacement and any deviations from the normal postoperative course. Implant malposition can result in spinal instability and postsurgical malalignment. Knowledge of the surgical approach (anterior, posterior, lateral, or caudal) is critical to interpretation of postoperative spines [13]. Transitional lumbosacral vertebral bodies can result in inaccurate identification of the pathologic level, necessitating correlation between radiological and surgical level labelling. Use of software spinal level labelling saved to picture archiving and communication system (PACS) images allows for correlation between the reported pathologic level and patients’ anatomy. The most common hardware placement complication is not placing hardware at the wrong level, but misangulated screw positioning and depth at the correct level.

**Fig. 2:**
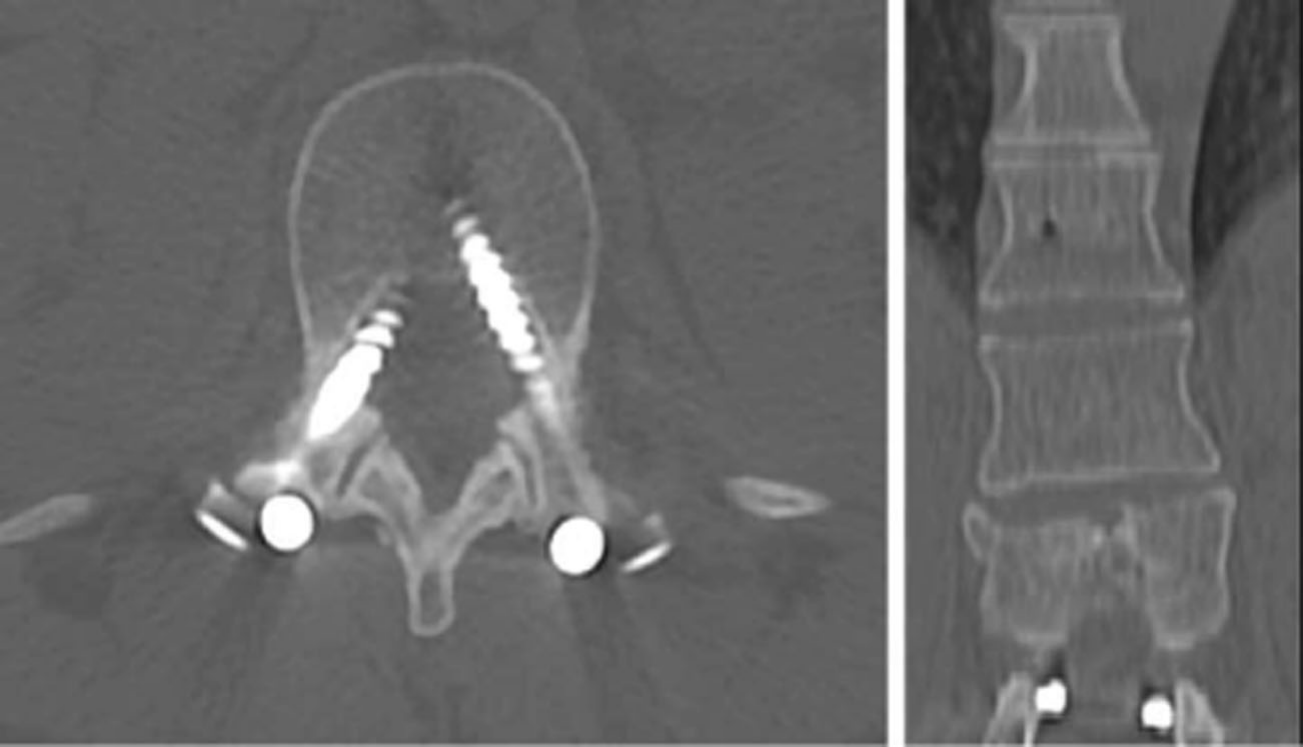
A 31-year-old female: Motor vehicle accident and compression fracture of T12: axial CT (**a**) and coronal reformats (**b**) show misplaced bilateral transpedicular screws at L1 abutting the medial cortex of pedicles

**Fig. 3:**
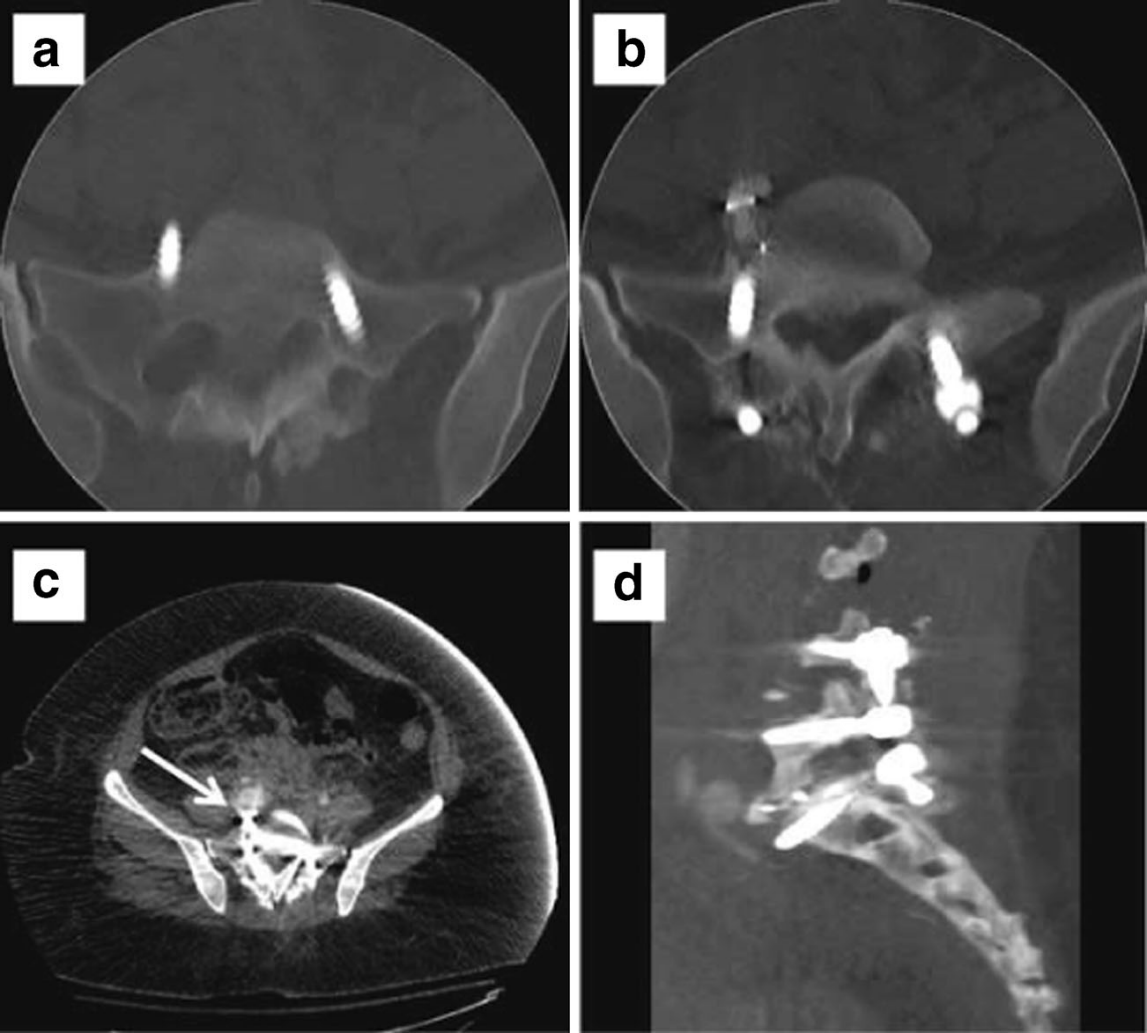
Status post L4–5 and L5–S1 fusion. Spacer cage is displaced anterolaterally to the right with a right S1 screw breaching the anterior cortex on axial CT images. **a** and **b** Axial CT slice through the lower abdomen shows retroperitoneal haemorrhage and right internal iliac artery pseudoaneurysm (*arrow*), abutting the displaced cage on sagittal reformats (**d**)

Anterior fixation screws should traverse the vertebral body without entering an adjacent endplate or the posterior vertebral body cortex. Posterior fixation screws should traverse the vertebral pedicle medially without disrupting the canalicular cortex, entering the neuroforamen or the anterior vertebral body cortex, while maintaining a parallel course to the endplate. Sacral screws may be anchored to the anterior cortex. A medial angulation of the screw course with violation of the medial cortex can injure or cause inflammation of nerve roots in the lateral recess [14] (Fig. 2). Cord ischaemia/infarct from aberrant radicular artery injury is rarely reported. Imaging guidance of pedicle screw placement has resulted in a significant decrease in perforation/cortical disruption through the use of computer navigation, at a rate of 6 % as compared to the 15 % with conventional freehand insertion. In a meta-analysis performed in 2012, 0 of 4184 screws placed by navigation resulted in neurological complication, while 3 of 3725 screws placed by freehand had neurological complications [15]. Among navigation-assisted screw placements, a higher accuracy has been reported with 3D as compared to 2D, 95.5 % versus 84.3 %, with 68.1 % for conventional fluoroscopy without navigation [16]. Interbody spacer position should be confirmed in both horizontal and vertical planes on serial imaging studies. Radiolucent spacers are fitted with radiopaque markers delineating the spacer position and malposition is radiographically suggested when the posterior marker is less than 2 mm anterior to the posterior vertebral body margin. Anterior malpositioning of sacral pedicle screws may irritate the L5 nerve roots, which are draped along the anterior sacral surface, resulting in acute L5 radiculopathy. Anterior malpositioning of lumbar pedicle screws causing injury to the iliac vessels can result in significant haemorrhage and is typically identified intraoperatively (Fig. 3). Complications related to prosthesis in total disc replacement (including migration, subsidence, implant failure, and endplate fractures) are reported in 2 to 39.3 % of patients [17].

### Postoperative collections

Postoperative fluid collection in the operative bed may represent seroma, CSF collection, haematoma, or abscess and can cause symptoms by compression of the thecal sac or nerve roots. Haematoma presents in hours to days following surgery, with the vast majority in the subcutaneous or epidural space. MR is able to distinguish haematoma from seroma better than CT by fluid signal characteristics; however, in many cases, the signal characteristics are not specific. Gradient sequences, useful for identifying haematomas elsewhere, are of little value in the setting of metallic hardware constructs because of extensive artefacts, as described earlier. MR is able to delineate the size of the fluid collection and whether it communicates with the spinal canal determining compression of the thecal sac. Compression of the thecal sac is a common finding, occurring in 58 % of cases reported by one study, none with new postoperative neurologic deficits. This study demonstrated that, on average, haematomas extended half a vertebral body beyond the decompression [18]. Less than 1 % of postoperative haematomas require evacuation for decompression [19]. However, neurologic recovery depends on the degree of deficit and the time of decompression; thus early diagnosis is critical [20]. Even with contrast enhancement, superinfection is difficult to assess, as there is extensive adjacent enhancing soft tissue and granulation tissue around all collections. Ultrasound plays an important diagnostic and therapeutic role for evaluation of subcutaneous collections, demonstrating septations that are occult on MRI. Use of bone morphogenetic proteins has been associated with increased incidence of seroma formation [21].

**Fig. 4:**
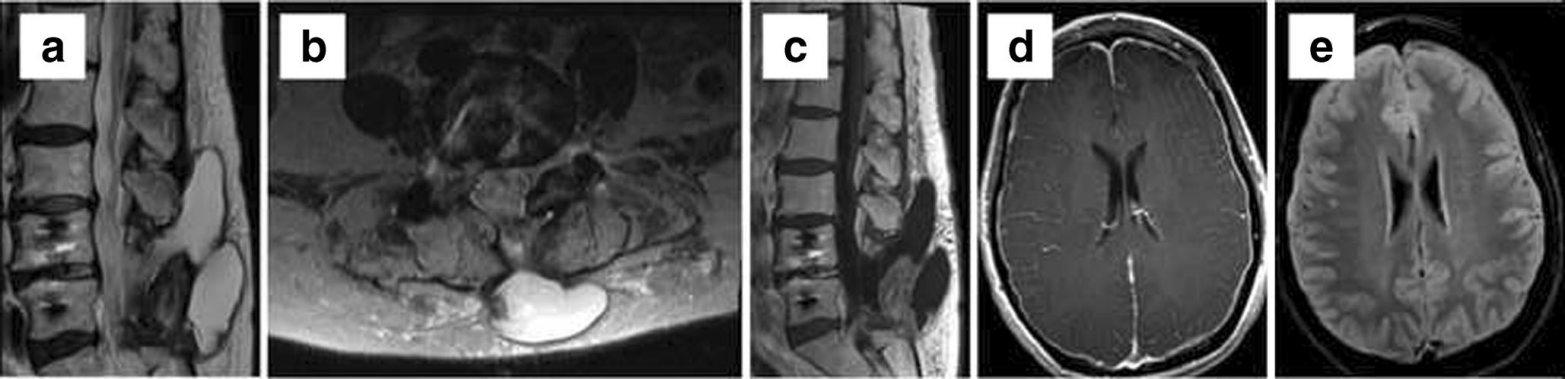
Sagittal and axial T2 WIs (**a** and **b**) show a large epidural and posterior paraspinal fluid collection in a patient with L4–5 decompression and fusion. Postcontrast image (**c**) shows no significant enhancement around the collection. The patient had orthostatic headaches and MRI brain confirmed dural thickening and effusion on FLAIR (**d**) with diffuse pachymeningeal enhancement (**e**) consistent with intracranial hypotension due to CSF leak and psedomeningocele formation

Incidental durotomy during lumbar spinal surgery can result in CSF leak with or without pseudomeningocele formation, CSF fistula, and nerve root herniation. Identification of CSF leaks is essential in preventing severe headaches from intracranial hypotension and the possibility of meningitis. Incidental durotomy and pseudomeningocele formation occur with an incidence depending on the extent of surgery. Pseudomeningocele formation occurs in 5.9 % of disectomy cases and 43 % of tethered spinal cord release cases [22]. Higher incidence of incidental durotomy is seen in revision surgeries [23]. Slight expansion of the dural sac into a surgical bony defect does not represent a pseudomeningocele [24]. CSF fistula has been reported to have an incidence of 2 % [25]. Imaging is performed with MR as first line modality and CT myelography if necessary (Fig. 4). Spine MRI may show epidural fluid collections and/or paraspinal fluid, dilatation of the epidural venous plexus, and diffuse dural thickening and enhancement [26]. Although nerve root herniation can occur secondary to dural tear during surgery, long-term follow-up has shown no significant differences in incidence for nerve root injury or functional disability.

MR brain imaging findings of subdural collections, enlarged dural sinuses, and parenchymal sagging are characteristic of intracranial hypotension from CSF leak [27]. Delayed/dynamic CT myelography can help identify slow leaks [28]. Dynamic myelography can differentiate a communicating CSF collection from a seroma. MR myelography, with off-label intrathecal gadolinium injection, has been shown to identify the CSF leak in one out of five patients with leaks occult to CT myelography [29]. Radionuclide myelography can help to detect slow, intermittent leaks.

Intracranial haemorrhage can occur post spine surgery, most commonly in the posterior fossa, and the aetiology is thought to be CSF leak and intracranial hypotension in patients with durotomy [30].

### Infection

**Fig. 5:**
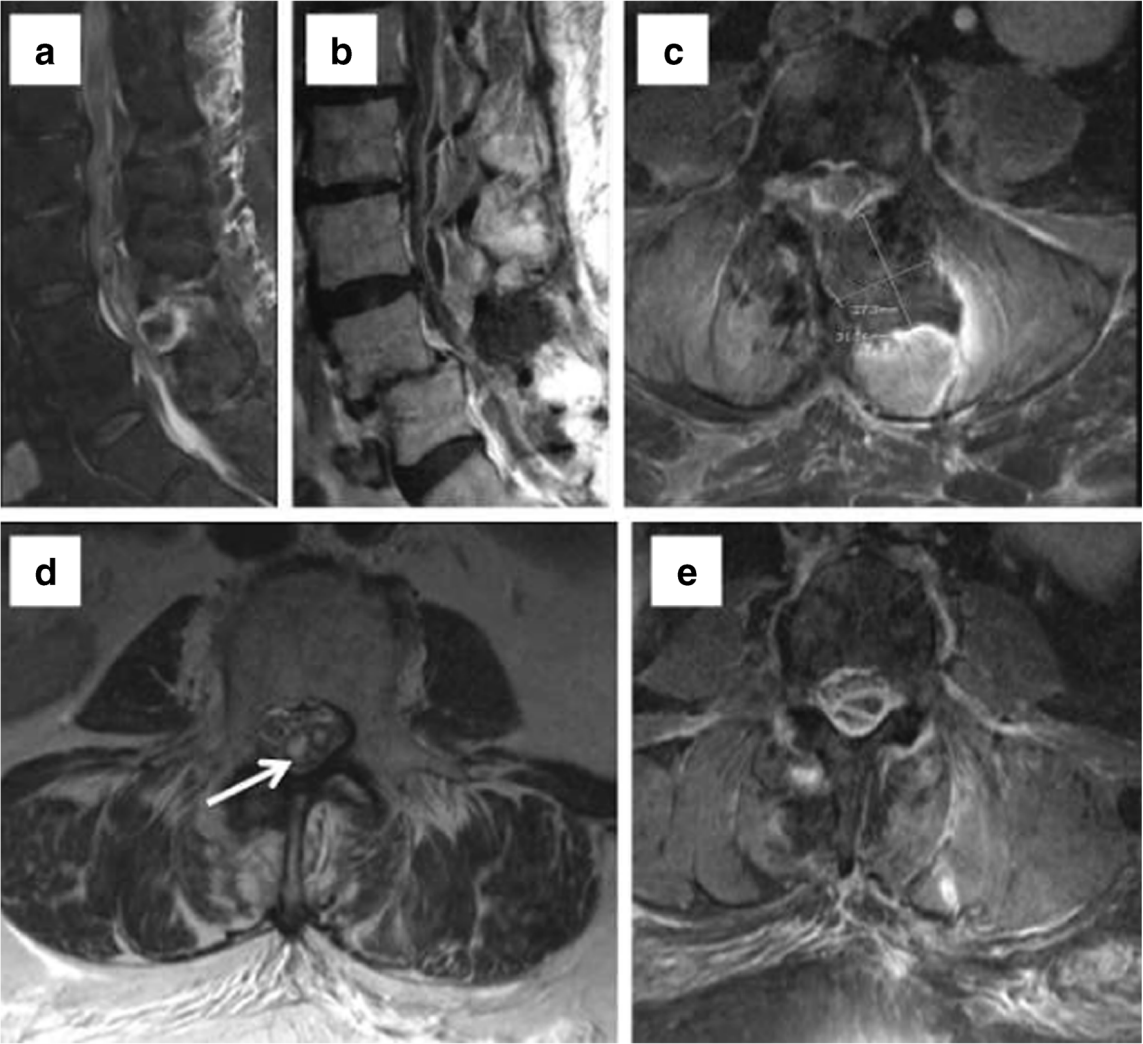
Sagittal T2 (**a**), sagittal postcontrast (**b**), axial postcontrast images (**c** and **e**), and axial T2 WI (**d**). Left laminectomy at L4 with peripherally enhancing collection at the operative site. Multiloculated, peripherally enhancing collections were seen in the dorsal epidural space (*arrow* in **d**) compressing the thecal sac. A multiloculated epidural abscess was drained at surgery

**Fig. 6:**
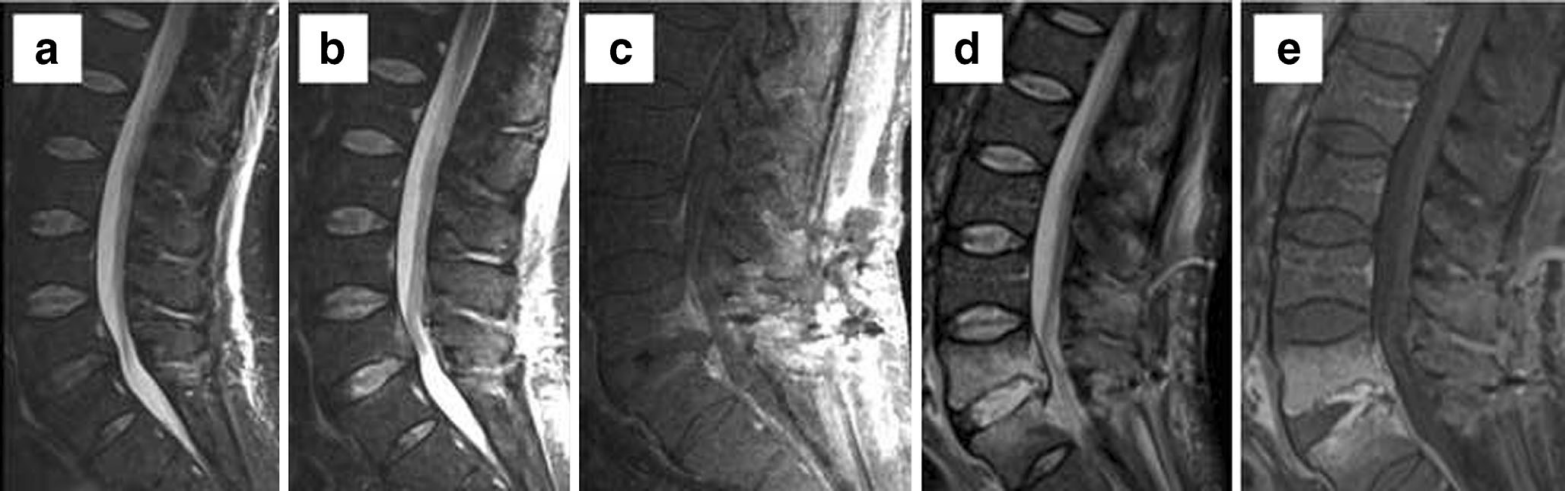
Evolution of changes over time post discectomy at L4–5. Sagittal STIR image (**a**) shows minimally increased T2 signal in the disc posteriorly and preserved marrow signal. Two-week follow-up MRI shows interval increased signal through most of the disc (**b**) with marrow oedema and enhancement on postcontrast images (**c**). Significant interval worsening was seen on further follow-up on both STIR (**d**) and postcontrast images (**e**) with epidural phlegmon

Infection in the early postoperative course is a result of direct contamination and haematogenous seeding or hardware-related inflammatory response in the late postoperative course. As with other lumbar spinal surgery complications, the incidence of infection is correlated with the extent of surgery [31, 32]. Minimally invasive surgery has an incidence of 0.4 % compared to 1.1 % for the traditional open approach [33]. Nerve root enhancement in the early postoperative period is not a specific finding for infection and frequently represents a transient sterile radiculitis. However enhancement persisting for more than 6 months is considered abnormal [34]. Enhancement of the posterior disc from an aseptic reaction is seen in the majority of the patients, which can mimic early infection. Vertebral endplate oedema and enhancement are normal postoperative changes in asymptomatic patients [35]. MR specificity for spondylodiscitis increases when there is vertebral body destruction, paraspinal/epidural phlegmon or abscess, and psoas muscle enhancement (Fig. 5). Spondylodiscitis will show increased T2 signal throughout the disc, while discectomy only at the curettage site [36] (Fig. 6).

Radionuclide dual tracer Tc-99 m sulphur colloid and In-111–labelled leukocyte scans have a sensitivity of 90 % in diagnosing infection [37]. Nuclear medicine Tc-99m MDP bone scans are of limited value given their low specificity [38]. When radionuclide studies are performed, SPECT/CT rather than planar/SPECT should be performed [39]. Given nonspecific imaging findings, clinically suspected postoperative spondylodiscitis is commonly evaluated with CT-guided percutaneous biopsy.

### Loosening

Loosening is defined radiographically as a lucent rim of 2 mm or greater surrounding the hardware, particularly when this lucency enlarges on sequential studies. It is best visualised on CT or plain radiographs. Loosening of vertebral body screws in older anterior constructs without locking screw plates may result in the backing out of the screw. Nuclear medicine bone scintigraphy demonstrates increased radiotracer uptake at sites of motion. Functional fusion, defined as less than 3° of motion between flexion and extension views performed 8–16 weeks postoperatively, depends on patient cooperation and can be underestimated by muscle guarding/spasm. Osseous fusion is demonstrated radiographically by bridging trabecular bone. Premineralised osteoid resulting in functional fusion is radiolucent, with radiographically evident fusion not evident until 6–9 months postoperatively [40]. Centrally interrupted trabeculation can suggest motion, delayed union, and/or early pseudoarthrosis. Radionuclide scintigraphy may suggest pseudoarthrosis or loosening with tracer uptake seen beyond a year postoperatively. Interbody implants appear to float in the early postoperative period, as morselised autographs are not visible on plain radiographs and do not indicate loosening.

**Fig. 7:**
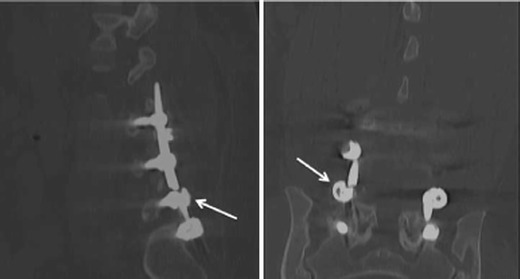
Sagittal and coronal CT reformats. Fracture of the vertical fusion rod with persistent anterolisthesis

**Fig. 8:**
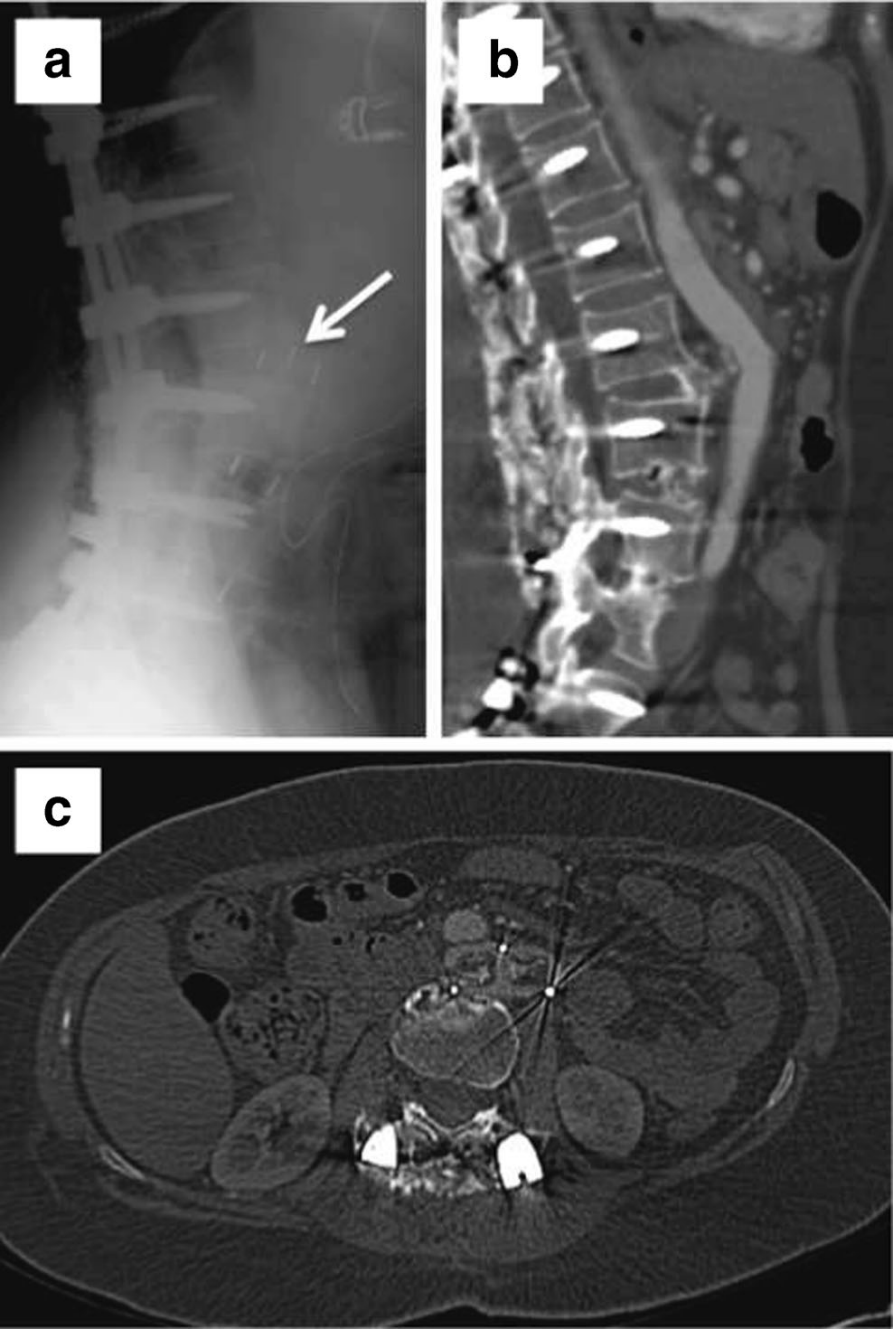
Lateral X-ray (**a**) shows anteriorly displaced spacer cage at L2–3. Sagittal (**b**) and axial CT (**c**) images show the cage indenting and displacing the abdominal aorta anteriorly better

**Fig. 9:**
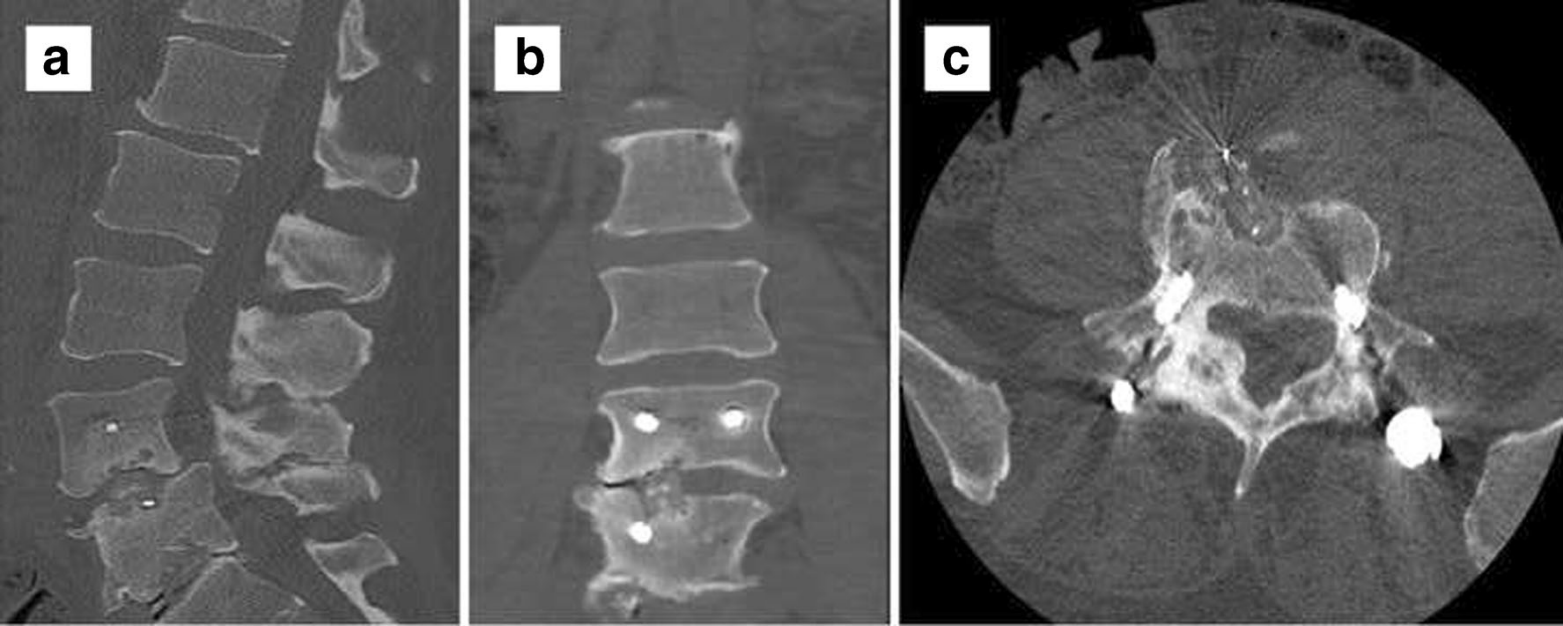
Sagittal, coronal, and axial CT images show migration of the fusion cage through the superior endplate of more than 3 mm resulting in loss of height restoration and subsidence

Hardware failure occurs when an implant fractures or is displaced in relation to adjacent osseous structures (Figs. 7 and 8). Failure is usually preceded by loosening due to persistent motion, pseudoarthrosis, or infection. Total disc replacement/arthroplasty carries complications of migration, displacement, subsidence, and endplate fractures. Bone graft extrusion was seen in 2 % of the cases in a study performed prior to the advent of titanium-threaded cage devices [41]. Although bone morphogenetic proteins enhance arthrodesis following spinal fusion, they have been shown to be associated with endplate resorption in 82 % of the patients following lumbar spinal surgery, resulting in cage subsidence in more than half of the cases [42]. Subsidence is defined as migration of the fusion cage through the osseous endplate of more than 3 mm resulting in loss of height restoration (Fig. 9). Imaging is able to assess for subsidence better as newer bioactive polyetheretherketone and carbon fibre cages cause significantly fewer artefacts than older stainless steel cages. Pedicle screws can fracture in 0.5 % of cases [43].

### New disease or disease progression

**Fig. 10:**
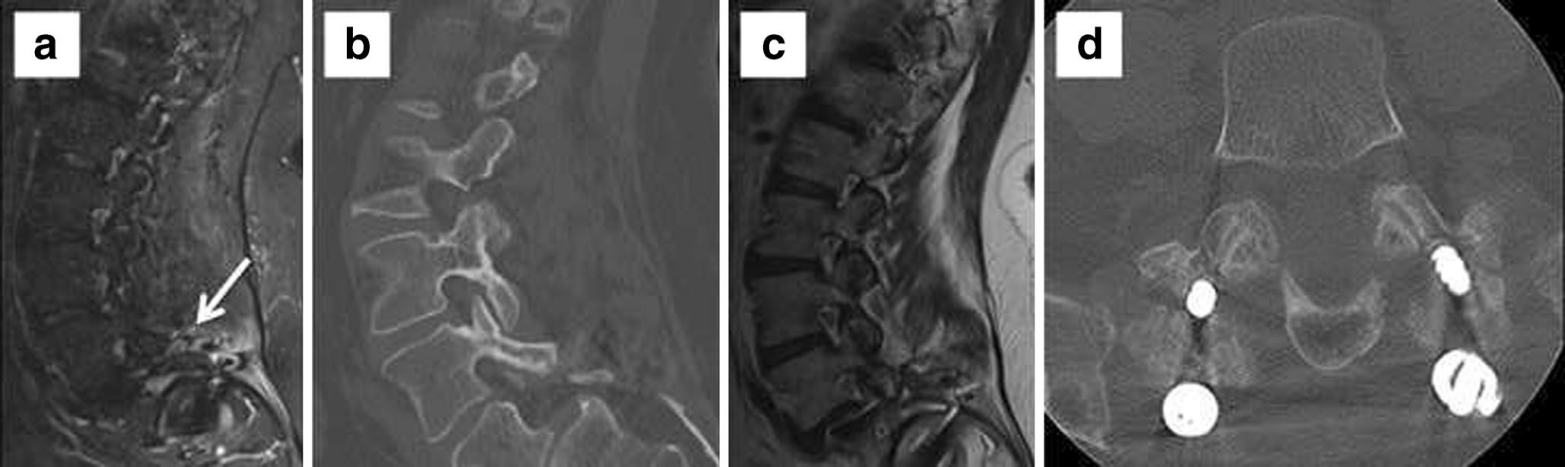
Pseudoarthrosis with lack of bony fusion on CT (**b** and **d**) and persistent increased signal on STIR and T2 WI (**a** and **c**) with worsening anterolisthesis

**Fig. 11:**
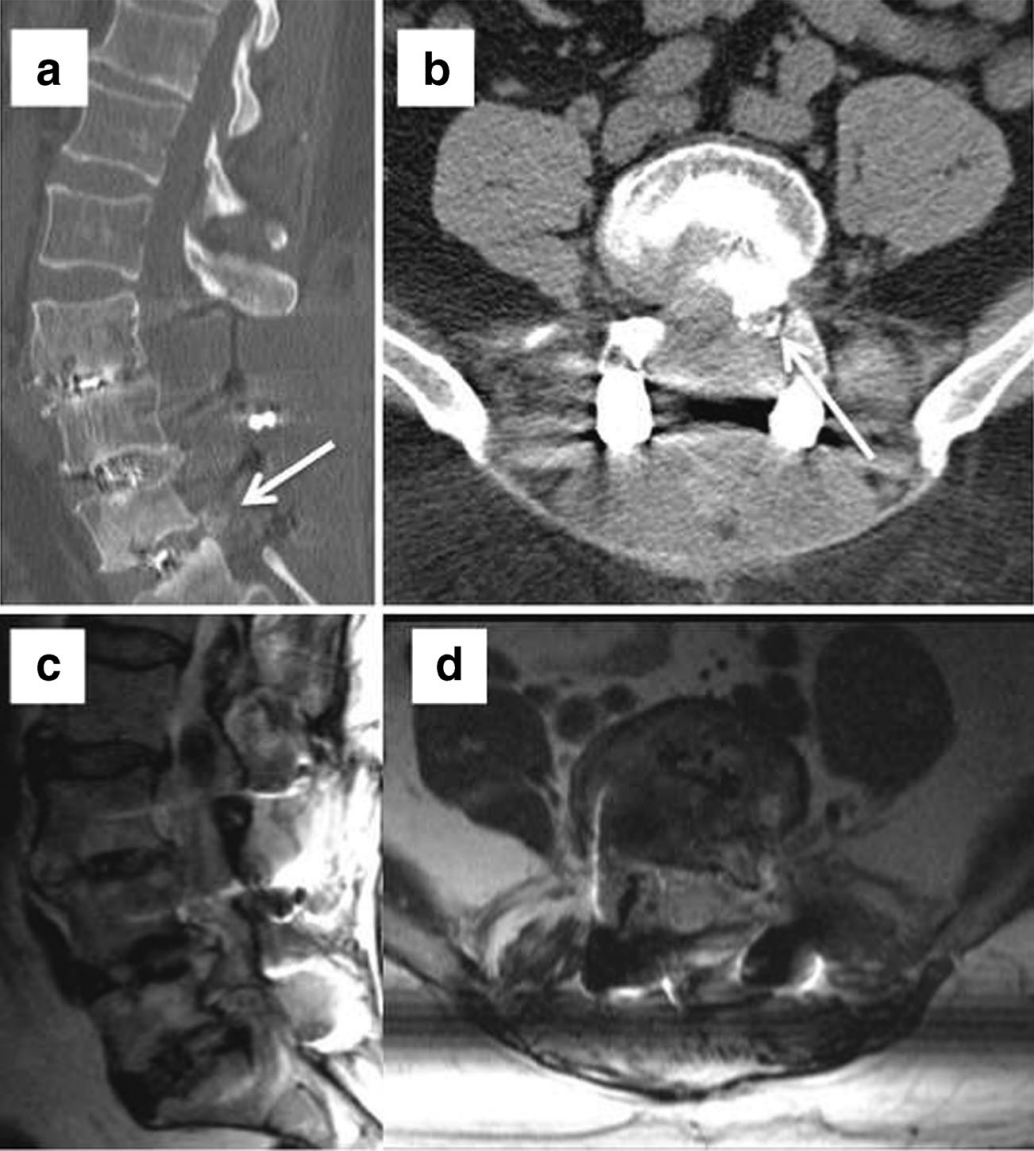
Heterotopic bone formation along the left ventrolateral aspect of the canal with left lateral recess stenosis is seen on CT (**a** and **b**) as well as sagittal and axial T2 WI (**c** and **d**)

Imaging plays an essential role in evaluating new diseases or disease progression caused by lumbar spinal surgery. Symptomatic new or recurrent disc herniation, peri-/epidural fibrosis, neuroforaminal stenosis, arachnoiditis, and radiculitis are key causes of FBSS. FBSS may result from both successful and unsuccessful osseous fusions. Successful lumbar spinal surgery with osseous fusion results in altered biomechanics with hypermobility of adjacent levels, causing accelerated degenerative changes. Unsuccessful osseous fusion can result in pseudoarthrosis. Pseudoarthrosis rates are higher for anterior than posterior fusions [44]. Findings of T1 hypointensity and T2 hyperintensity persisting between the vertebral body and bone graft for more than 6 months are suggestive of pseudoarthrosis (Fig. 10). Heterotopic bone formation, which is increased with the use of osteobiologics, is best assessed on CT. It occurs in the ventrolateral epidural space after transforaminal lumbar interbody fusion (TLIF) and facet complex after posterior lumbar interbody fusion (PLIF), resulting in central canal or foraminal stenosis, respectively. The degree of stenosis is best assessed on MRI (Fig. 11).

**Fig. 12:**
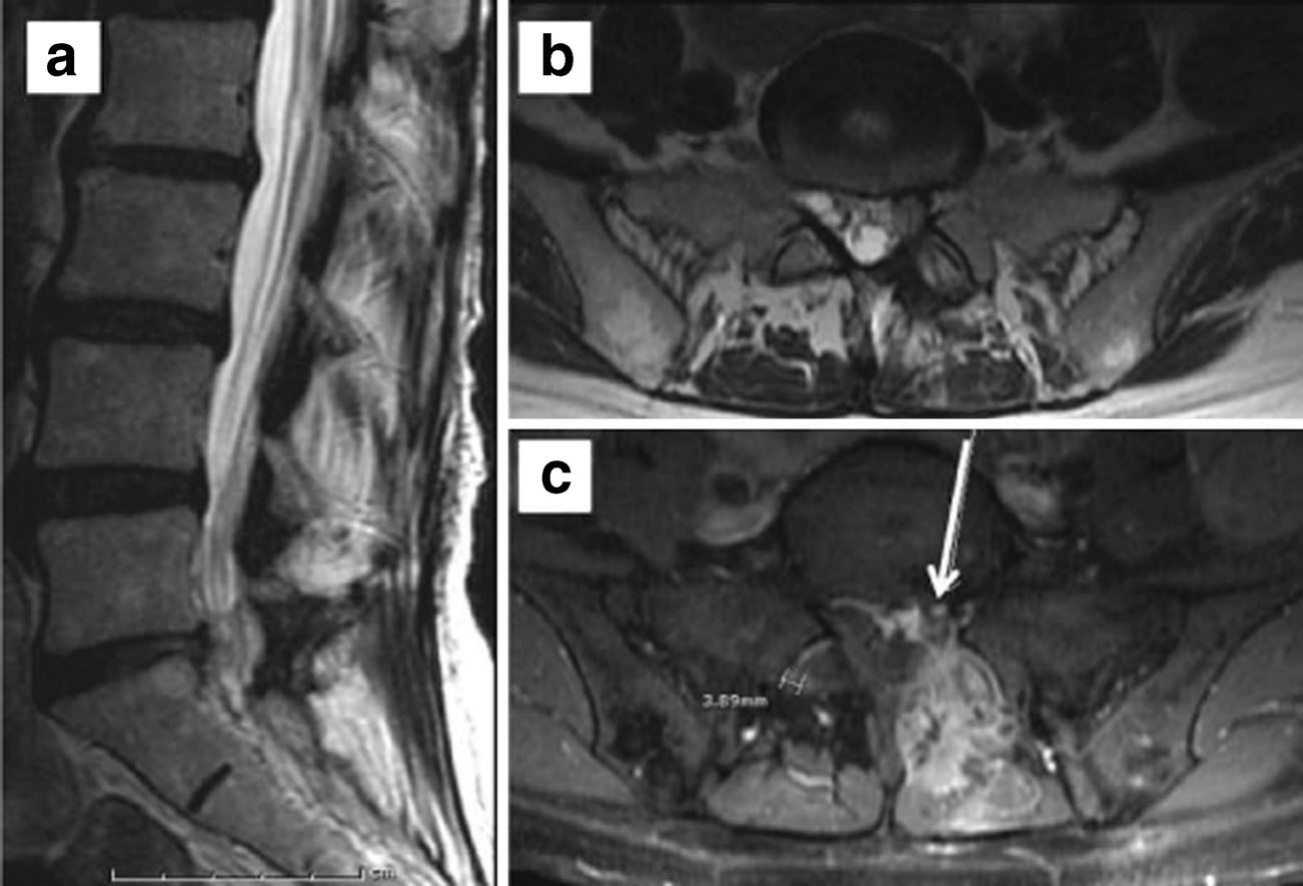
Patient with L5–S1 laminectomy and discectomy. Sagittal and axial T2 WIs (**a** and **b**) show hypointense soft tissue in the left lateral recess. Postcontrast image (**c**) shows a centrally nonenhancing disc surrounded by more infiltrating, enhancing granulation tissue along the left lateral aspect of the canal and left lateral recess

Recurrent disc herniations, defined clinically as recurrence after a 6-month pain-free postoperative period, varies between 3 and 18 % post lumbar discectomy in retrospective studies [45]. Imaging prospectively over a 2-year postoperative period demonstrated recurrent disc herniations in 23 % of the patients, half being asymptomatic [46]. MR imaging with contrast is the modality of choice because of superior contrast resolution. Contrast administration is key in distinguishing recurrent disc herniation from peridural fibrosis, with disc herniations demonstrating early central nonenhancement and peripheral enhancement due to granulation tissue or dilated epidural venous plexus, whereas fibrosis demonstrates diffuse enhancement. Delayed postcontrast imaging should be avoided in the postoperative spine as contrast may diffuse into disc material [47]. Discernment between recurrent herniation and fibrosis can be muddled when disc herniations contain central enhancing granulation tissue (Fig. 12). Additional features such as intermediate signal with irregular margins favour epidural fibrosis and low signal with smooth margins favours recurrent herniation.

Assessment of new diseases or disease progression should not be performed in the immediate postoperative period. Early postdisectomy changes with annulus fibrosis, epidural space oedema, and granulation tissue can resemble the preoperative appearance in 80 % of the patients [48]. Even when there is residual or recurrent herniation, this may be stable and asymptomatic or spontaneously regress [34]. Similarly, epidural fibrosis is frequently asymptomatic. Some authors have described epidural fibrosis as a radiological entity independent of clinical symptoms, while others have concluded that diffuse epidural scarring correlates to symptoms, but small focal scarring does not [49, 50]. Epidural scarring may be occult on MR, evident only on epiduroscopy; thus epiduroscopy may be considered to evaluate for fibrosis in symptomatic patients with negative MR findings [51]. Discernment between fibrosis and recurrent herniation is of clinical value as reoperation may be beneficial in recurrent herniation but not fibrosis.

**Fig. 13:**
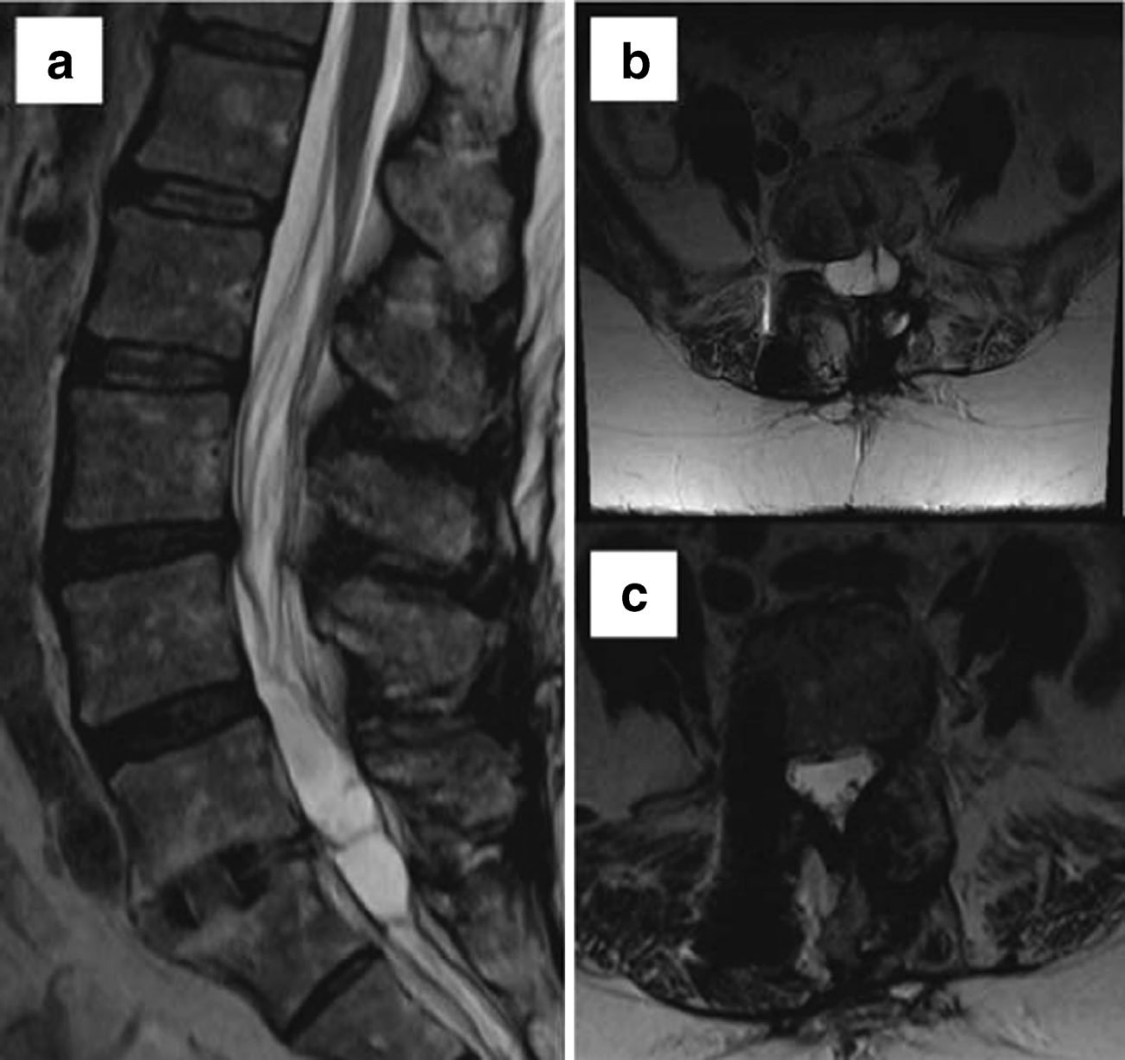
Status post L5–S1 fusion. Sagittal (**a**) and axial (**b** and **c**) T2 WI: redundant and thickened cauda equina nerve roots arranged along the periphery of the thecal sac with meningeal diverticulae

Sterile arachnoiditis is a cause of persistent pain in 6–16 % of postsurgical cases [24]. Arachnoiditis has a spectrum of MR imaging findings with variable enhancement of clumped nerve roots, an “empty” thecal sac with the nerve adhered to the dural walls, or mass-like filling of the thecal sac (Fig. 13).

**Fig. 14:**
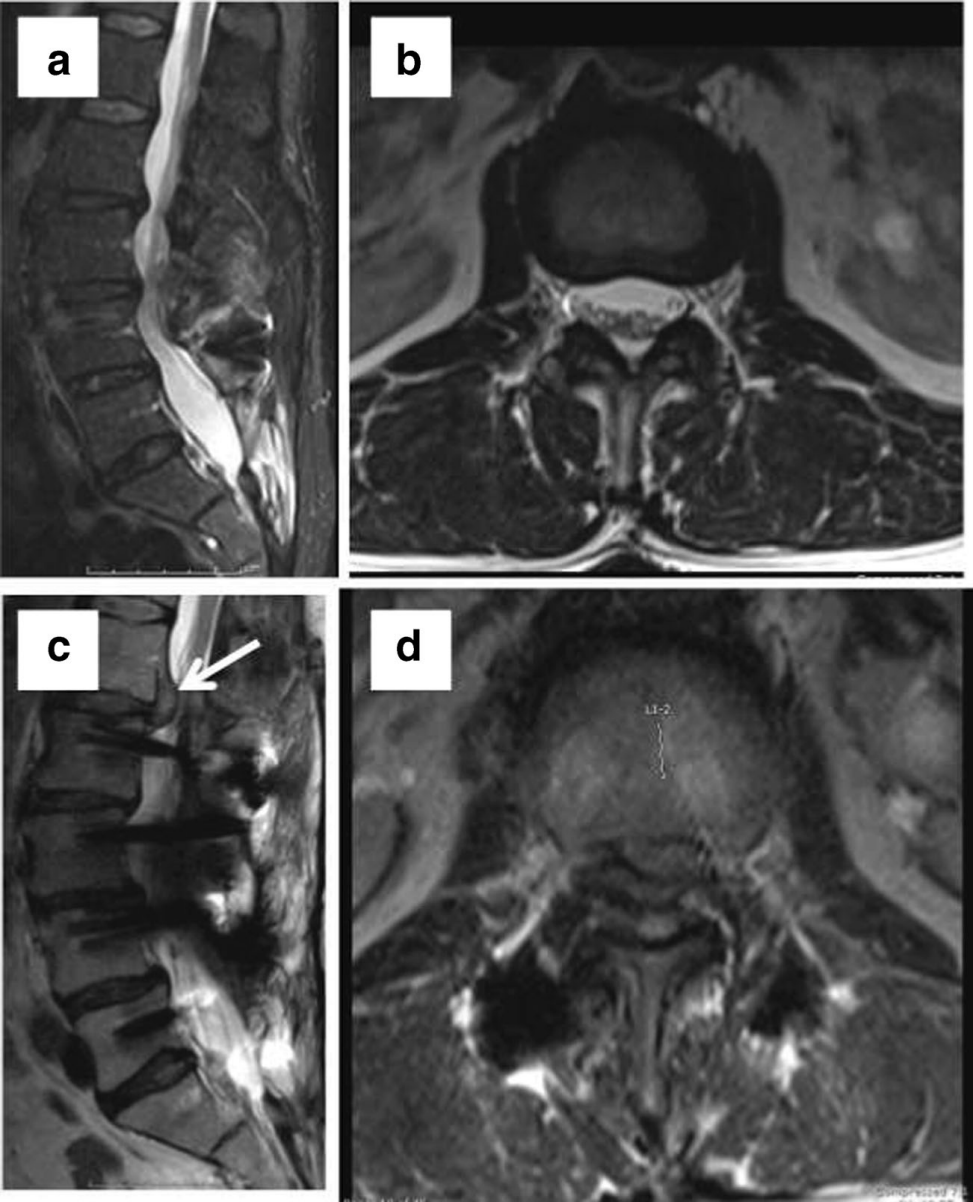
Status post L2–L5 fusion. Worsening of degenerative changes with disc extrusion at the level just above fusion at L1–2 (**c** and **d**) compared to initial images done a year earlier (**a** and **b**)

Discectomy without height restoration results in neuroforaminal narrowing, which may worsen over an extended time period from altered stresses and motion resulting in facet hypertrophy. Clinically significant accelerated degenerative changes occur at a rate of 0.6–3.9 % per year following lumbar fusion, most often at the cranial perisurgical level [45, 52] (Fig. 14).

## Conclusions

Lumbar spine surgery is being increasingly performed and imaging evaluation of the postoperative spine has evolved significantly in the past decade. Advances in CT and MRI with reduction of image degradation due to hardware-related artefacts have improved the evaluation of the postoperative spine and early detection of complications.
